# A Rare Case of Lance-Adams Syndrome: Status Post-Successful Cardiopulmonary Resuscitation

**DOI:** 10.7759/cureus.32604

**Published:** 2022-12-16

**Authors:** Ali Rahman, Sura Alqaisi, Beth Helfman

**Affiliations:** 1 Internal Medicine, Northwell health at Mather Hospital, Port Jefferson, USA; 2 Internal Medicine, Memorial Healthcare, Pembroke Pines, USA; 3 Cardiology, Memorial Healthcare, Pembroke Pines, USA

**Keywords:** antiepileptic drugs, electroencephalography (eeg), intention myoclonus, myoclonic status epilepticus (mse), chronic posthypoxic myoclonus (pmh), lance-adams syndrome (las)

## Abstract

Lance-Adams syndrome (LAS), also known as chronic post-hypoxic myoclonus (PHM), is a rare condition that may present with intention myoclonus in a patient who has regained consciousness after cardiorespiratory arrest. This case report describes a patient who received successful cardiopulmonary resuscitation (CPR) after going into cardiac arrest. And regaining consciousness, the patient developed myoclonic jerks diagnosed as LAS. The patient responded well to treatment with clonazepam and physical rehabilitation.

## Introduction

Lance-Adams syndrome (LAS), also known as chronic post-hypoxic myoclonus (PHM), is a rarely occurring condition that may present with intention or action myoclonus within days or weeks of regaining consciousness following hypoxic episodes, such as cardiorespiratory arrest. The condition can be easily misdiagnosed since its clinical features may overlap with acute PHM and typically present in comatose patients within the first 24 hours of cardiac arrest. The prognosis of LAS is generally favorable, and patients usually respond well to treatment with antiepileptic drugs (AEDs) and rehabilitation measures.

LAS is a rare complication, with less than 150 cases reported in the literature. In this report, we present a case of a patient who developed LAS after receiving cardiopulmonary resuscitation (CPR) for sudden cardiac arrest.

## Case presentation

A 54-year-old man with a past medical history of hypertension presented to the emergency department of our hospital with shortness of breath and cough. The patient was hypoxic and was noted to have abdominal distension on examination. An electrocardiogram (EKG) was obtained, which revealed atrial fibrillation with a rapid ventricular response. His laboratory results were significant for an increase in white blood cell count (Table 1). The patient was started on enoxaparin and diltiazem and was transferred to the intensive care unit (ICU). Bilevel-positive airway pressure (BiPAP), diuresis, and intravenous beta-blocker therapy were also initiated. After improving the patient’s heart rate and ventilatory status, he was moved back to the medicine floor.

**Table 1:** Laboratory results WBC, white blood cell; BUN, blood urea nitrogen; ESR, erythrocyte sedimentation rate; SAR-COV-2, severe acute respiratory syndrome coronavirus 2; PCR, polymerase chain reaction

Labs	Values	Normal range	Units
WBC	15.3	4.5-11	10^9^/L
Hemoglobin	14.4	12-16	g/dL
Platelets	160	130-400	10^9^/L
Neutrophils	85	40-60	%
Lymphocytes	8.2	20-40	%
Monocytes	5	1.7-9.3	%
Eosinophils	1	0-5	%
Basophils	0.8	0-3	%
Sodium	137	137-145	mmol/L
Potassium	4	3.5-5.2	mmol/L
Chloride	99	98-107	mmol/L
Carbon dioxide	22	22-30	mmol/L
BUN	11	7-17	mg/dL
Creatinine	0.9	0.52-1.04	mg/dL
ESR	213	0-29	mm/hour
D-dimer	100	<250	ng/mL
Ferritin	74	12-50	ng/mL
Fibrinogen	220	200-400	mg/dL
SAR-CoV-2 PCR	Negative	Negative	

While on the medicine floor, the patient suddenly went into cardiac arrest and was found to have pulseless electrical activity. CPR was immediately started, and endotracheal intubation was performed. The patient had a successful return of spontaneous circulation after 15 minutes of CPR. He was shifted again to the ICU, where he persistently remained hypotensive and had to be started on intravenous vasopressors. An echocardiogram was performed, which demonstrated a reduced ejection fraction (EF) of 40%. A few days later, the patient was started on a spontaneous breathing trial to wean off of mechanical ventilation. He was eventually extubated, and intravenous vasopressors were stopped.

**Figure 1:**
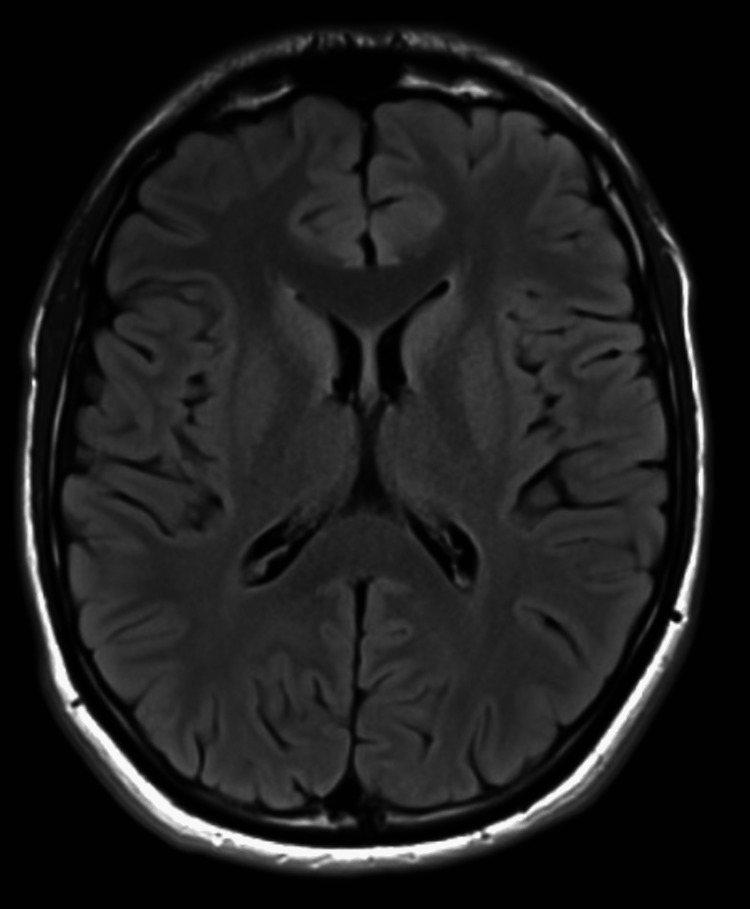
Brain MRI with no abnormal signal intensity

**Figure 2:**
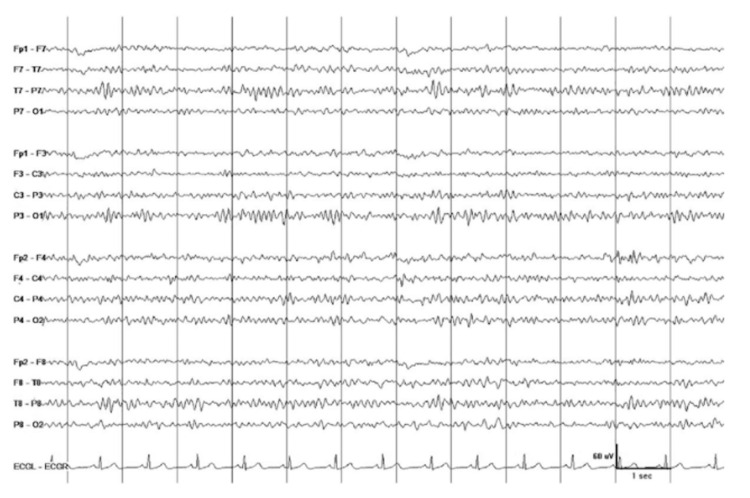
Electroencephalogram

Following this, the patient was downgraded to the medical floor, where he was noted to have jerky, myoclonic movements of his face and limbs, which lasted for 1-2 minutes; it was stimulus sensitive whenever the patient asked to initiate movements such as holding a cup, moving his arms, or lifting his legs. The rest of his neurological examination was unremarkable, including full consciousness and intact sensation, with no signs of meningeal irritation or cerebellar dysfunction. The neurology department was consulted, and magnetic resonance imaging (MRI) of the brain and electroencephalogram (EEG) were performed. MRI scan of the brain did not show any acute findings (Figure 1); however, the EEG was mildly abnormal and showed some mild, diffuse theta slowing, which was suggestive of mild, diffuse cerebral dysfunction (Figure 2). The patient was diagnosed with LAS based on the development of action myoclonus following a successful CPR. He was started on treatment with clonazepam along with physical therapy, which resulted in an improvement in his symptoms over two weeks.

## Discussion

Myoclonus refers to sudden, brief, jerky, involuntary movements of the muscles. Positive myoclonus is caused by sudden muscle contractions, while negative myoclonus is caused by brief lapses in muscle tone. Myoclonus can be classified based on the etiology, pathophysiology, and anatomic site of origin, and can be a sign of a wide variety of neurological disorders, such as Creutzfeldt-Jakob disease, Parkinson’s disease, Huntington’s disease, Alzheimer’s disease, and multiple sclerosis [1]. PHM is a type of myoclonus that may be seen in patients after hypoxic episodes, for example, after successful CPR in survivors of cardiorespiratory arrest [2].

PHM can be divided into two subtypes, acute and chronic, depending on the time of occurrence of myoclonic jerks after hypoxia. Acute PHM, also known as post-hypoxic myoclonic status epilepticus (MSE), is a stimulus-sensitive and spontaneous myoclonus that usually develops within the first 24 hours after hypoxic brain injury in deeply comatose patients. Acute PHM has been found to occur in around 19-37% of patients after successful CPR. It can be cortical and/or subcortical in origin and is associated with poor neurofunctional recovery in most cases. Hence, acute PHM is considered to be an indicator of poor prognosis [3]. As opposed to acute PHM, chronic PHM, also called Lance-Adams syndrome, is characterized by the development of action myoclonus days or weeks after a hypoxic episode. The patients usually regain consciousness by this time and are not comatose. LAS was first described by Lance and Adams in 1963, who reported four cases of intention or action myoclonus along with dysarthria and ataxia, which appeared a few days after cardiac arrest [4,5]. Since 1963, many cases of LAS have been described, and according to a systematic literature review published in 2016, around 163 cases have been reported in the literature worldwide [6].

An accurate distinction between MSE and LAS is important as the clinical characteristics and prognosis of these conditions are different. The timing of the onset of myoclonus after a hypoxic episode has traditionally been used to distinguish the two conditions. MSE usually presents within the first 12 hours of hypoxia and disappears after 48 hours in most cases. LAS, on the other hand, typically develops later and may run a chronic course [7]. However, the timing of onset is not a reliable feature to differentiate between the two subtypes of PHM, as LAS has been found to develop within hours of the hypoxic insult in some cases, thus complicating the diagnosis of acute versus chronic PHM [8]. The pattern of involvement of body parts may also not be a helpful distinguishing feature, as both subtypes may present with generalized or multifocal myoclonus, depending on whether the myoclonus is from a cortical or subcortical source [8,9]. Therefore, the presence or absence of coma is considered to be the single most important feature to distinguish between the two subtypes, with the presence of coma being a prerequisite for the diagnosis of MSE. In contrast, LAS is usually seen in patients who are conscious and aware. However, it is important to remember that LAS may easily be mixed up with MSE in patients who have regained consciousness but are receiving deep sedation, as this may make the patients appear comatose, similar to those with MSE [7,8].

The exact pathophysiology of LAS remains poorly understood; however, the literature suggests that PHM may originate from cortical and/or subcortical structures and may be associated with the depletion of serotonin or 5-hydroxytryptamine (5-HT) in the inferior olivary nucleus. Serotonin from the brainstem acts on the postsynaptic 5-HT receptors on the inferior olivary nucleus to decrease the magnitude of low-threshold calcium conductance, thus inhibiting its rhythmic discharge. The depletion of serotonin causes the neurons in the inferior olivary nucleus to remain in a state of unconstrained irritability, which may be responsible for the development of myoclonus after hypoxia. On the other hand, gamma-aminobutyric acid (GABA) has been found to inhibit neuronal excitability and, thus, reduce the incidence of PHM. This is accomplished through the action of GABA on presynaptic GABA type A receptors, where it inhibits the release of neurotransmitters, such as glutamate, or via its action on postsynaptic receptors that results in the opening of chloride channels and hyperpolarization [10,11].

Imaging modalities, such as computed tomography (CT) or MRI, may not be helpful in establishing a diagnosis of LAS. A review of cerebral MRIs performed in 12 patients with LAS after a mean of 2.5 years following the hypoxic event showed variable findings. Among the 12 patients, four did not have any abnormalities on MRI, four had nonspecific abnormalities such as mild cortical and cerebellar atrophy, three had cerebral infarcts, and one had focal infarcts in the right cerebellar hemisphere. A serial MRI study evaluating LAS in the early stages found that patients with LAS had early and transient involvement of the cerebellum and thalamus [12]. In our patient, the brain MRI did not reveal any acute changes. Other neuroimaging studies, such as single-photon emission computed tomography (SPECT) and positron emission tomography (PET), have been shown to provide insight into the pathophysiology of LAS. For instance, cerebral SPECT scans have been reported to show a reduction in perfusion in multiple areas of the brain, including the frontal lobe and the left temporal lobe. Similarly, PET scans have shown increased glucose metabolism in the mesencephalon, ventrolateral thalamus, and pontine tegmentum in patients with LAS [12,13].

Neurophysiological studies, such as electroencephalography (EEG), electromyography (EMG), and somatosensory evoked potentials (SSEPs), have also been used for the evaluation of PHM [6,14]. These studies have been found to be particularly useful in delineating the subtypes of PHM by differentiating between cortical and subcortical myoclonus and diffuse versus focal epileptiform activity. EEG is considered to be the most reliable tool for evaluating PHM and can aid in the diagnosis of LAS without the use of clinical features. According to a recent review, EEGs in one-third of the patients with LAS showed epileptiform activity, particularly within hours after cardiac arrest. Spike or polyspike-wave discharges were seen primarily at the vertex in half of these patients. Some of the patients had diffuse or focal slowing on the EEG, as was the case with our patient. Finally, around 20% of the patients were found to have a normal EEG. EEG-EMG polygraphy revealed jerk-locking in around 60% of the cases, while normal-sized and giant SSEPs were noted in an equal number of patients [6,8].

Due to the rarity of LAS, no established treatment guidelines are available, and different medications based on the proposed pathophysiology of this condition have been used [13]. At present, the goal of treatment is to increase serotonin levels and decrease the levels of excitatory amino acid neurotransmitters, such as glutamate. AEDs that can increase the levels of serotonin in the brain, such as clonazepam and sodium valproate, are considered to be the first-line treatment options to control post-hypoxic myoclonic seizures [10,15]. Medications, including lamotrigine, riluzole, and tetrahydro-nicotinic acid, which cause a decrease in the excitatory amino acid neurotransmitters, have also been found to be helpful in the treatment of LAS. According to some studies, subcortical PHM is usually treated with clonazepam, while levetiracetam or piracetam can often be used to treat cortical myoclonus following hypoxia [10,14,15]. In addition to drug therapy, rehabilitation interventions, such as gait and basic activities of daily living (ADL) training, have also been reported to slow the progression of LAS and decrease the chances of developing additional disabilities [16].

Our patient, who went into sudden cardiac arrest, regained consciousness after successful CPR. The patient later developed intention myoclonus and was found to have mild, diffuse slowing on the EEG. A diagnosis of LAS was established in a timely manner based on his clinical features and EEG findings. He was promptly started on clonazepam therapy along with physical therapy and showed significant clinical improvement. This case report highlights the fact that LAS is a rarely occurring condition; however, in patients who develop uncontrolled myoclonus after regaining consciousness following successful CPR, the possibility of LAS should be strongly considered. Failure to recognize the condition in a timely manner may lead to a delay in treatment and/or the initiation of inappropriate drug therapy. In contrast, timely diagnosis and initiation of treatment at an early stage of the disease can significantly improve the quality of life in these patients [17].

## Conclusions

LAS, or chronic PHM, is a rare neurological disorder that may present with myoclonic jerks within days or weeks of cardiorespiratory arrest. Patients are usually conscious and aware of when the symptoms start, and this feature can help distinguish this condition from acute PHM, which typically develops in deeply comatose patients. EEG findings may also help establish a diagnosis. A combination of AEDs and rehabilitation interventions can be used to treat LAS. Patients usually have a favorable prognosis; however, the timely initiation of treatment is of great significance.

## References

[1] Ibrahim W, Zafar N, Sharma S (2021). Myoclonus. StatPearls [Internet].

[2] Zhang YX, Liu JR, Jiang B (2007). Lance-Adams syndrome: a report of two cases. J Zhejiang Univ Sci B.

[3] Bouwes A, van Poppelen D, Koelman JH (2012). Acute posthypoxic myoclonus after cardiopulmonary resuscitation. BMC Neurol.

[4] Lee HL, Lee JK (2011). Lance-Adams syndrome. Ann Rehabil Med.

[5] LA JW, AD RD (1963). The syndrome of intention or action myoclonus as a sequel to hypoxic encephalopathy. Brain.

[6] Freund B, Sutter R, Kaplan PW (2017). Lance-Adams syndrome in the pretargeted temperature management era. Clin EEG Neurosci.

[7] English WA, Giffin NJ, Nolan JP (2009). Myoclonus after cardiac arrest: pitfalls in diagnosis and prognosis. Anaesthesia.

[8] Freund B, Kaplan PW (2017). Post-hypoxic myoclonus: Differentiating benign and malignant etiologies in diagnosis and prognosis. Clin Neurophysiol Pract.

[9] Acharya JN (2017). Post-hypoxic myoclonus: the good, the bad and the ugly. Clin Neurophysiol Pract.

[10] Guo Y, Xiao Y, Chen LF, Yin DH, Wang RD (2022). Lance Adams syndrome: two cases report and literature review. J Int Med Res.

[11] Muddassir R, Idris A, Alshareef N, Khouj G, Alassiri R (2021). Lance Adams syndrome: a rare case presentation of myoclonus from chronic hypoxia secondary to COVID-19 infection. Cureus.

[12] Waddell A, Dirweesh A, Ordonez F, Kososky C, Reddy Peddareddygari L, Grewal RP (2017). Lance-Adams syndrome associated with cerebellar pathology. J Community Hosp Intern Med Perspect.

[13] Nigam GB, Babu SS, Peter CS, Peter CS (2016). Lance-Adams syndrome: a special case of a mother. Indian J Crit Care Med.

[14] Gupta HV, Caviness JN (2016). Post-hypoxic myoclonus: current concepts, neurophysiology, and treatment. Tremor Other Hyperkinet Mov (N Y).

[15] Marcellino C, Wijdicks EF (2020). Posthypoxic action myoclonus (the Lance-Adams syndrome). BMJ Case Rep.

[16] Polesin A, Stern M (2006). Post-anoxic myoclonus: a case presentation and review of management in the rehabilitation setting. Brain Inj.

[17] Shin JH, Park JM, Kim AR, Shin HS, Lee ES, Oh MK, Yoon CH (2012). Lance-Adams syndrome. Ann Rehabil Med.

